# Bone marrow toxicity in patients with locally advanced cervical cancer undergoing multimodal treatment with VMAT/IMRT: are there dosimetric predictors for toxicity?

**DOI:** 10.1186/s40001-024-02041-w

**Published:** 2024-08-31

**Authors:** D. Hallqvist, C. Kormann, S. Pigorsch, M. Kiechle, S. E. Combs, D. Habermehl

**Affiliations:** 1 Department of Radiation Oncology, Klinikum rechts der Isar, School of Medicine, Technical University of Munich (TUM), Munich, Germany; 2 Department of Obstetrics and Gynecology, Klinikum rechts der Isar, Technical University of Munich (TUM), Munich, Germany; 3 Department of Radiation Sciences (DRS), Institute of Radiation Medicine (IRM), HelmholtzZentrum München, Neuherberg, Germany; 4 Deutsches Konsortium für Translationale Krebsforschung (DKTK), Partner Site Munich, Munich, Germany; 5 Department of Radiation Oncology, Giessen-Marburg University Hospital, Giessen, Germany

**Keywords:** Advanced cervical cancer, Chemoradiation, Bone marrow, Acute hematological toxicity

## Abstract

**Purpose:**

For women with locoregionally advanced cervical cancer, the standard of care treatment is the curatively intended chemoradiation therapy (CRT). A relationship between bone marrow (BM) dose–volume histograms (DVHs) and acute hematological toxicity (HT) has been debated recently. Aim of this study was the evaluation of BM dose constraints and HT in a contemporary patient cohort.

**Methods:**

Radiation treatment plans of 31 patients with cervical cancer (FIGO stage IIB–IVB) treated with intensity-modulated radiotherapy and simultaneous chemotherapy were explored retrospective. Pelvic bones (PB) and femoral heads (FH) were contoured and DVHs were correlated with white blood cells (WBC), hemoglobin levels and platelets.

**Results:**

Comparing the absolute blood levels with the dose volumes of both FH and PB the data showed a significant correlation between WBC and the median dose of the FH and the median dose, V_30Gy_, V_40Gy_ and V_50Gy_ of the PB. A correlation between the toxicity grade of anemia and mean dose, maximum dose and V_5Gy_ of the PB was found. Counting the highest grade of HT of all three blood levels of each patient, significant correlations were found for the mean and median dose, V_30Gy_, V_40Gy_ and V_50Gy_ of the PB.

**Conclusion:**

The results show that blood levels may correlate with distinct dosimetric subvolumes of critical bone marrow compartments with a potential impact on therapeutic outcome and treatment-related toxicity. The data presented are in line with the previous findings on the relevance of dosimetric exposure of pelvic bony subvolumes.

## Introduction

For women with loco-regionally advanced cervical cancer FIGO stage IIA–IVA the current standard of care treatment is pelvic external beam radiotherapy and concurrent cisplatin-based chemotherapy [1–3]. Acute and late toxicity remain a challenge and are still common in multimodal therapy protocols. Furthermore, incidental dose deposition in the adjacent pelvic and vertebral bone structures may contribute to chemotherapy-related bone marrow (BM) suppression [4]. Both, radiotherapy (RT) and chemotherapy (CT) are responsible for BM damage and may cause leukopenia, which may represent a severe dose-limiting side effect [5–7]. With the implementation of intensity-modulated RT (IMRT) and volumetric-modulated arc therapy (VMAT) the applied dose to pelvic organs at risk can be reduced and subsequently lead to fewer side effects in the irradiation of pelvic tumors [5, 8–10].

The purpose of this study was to examine a correlation between hematotoxicity (HT) and dose–volume parameters of irradiated BM in cervical cancer patients who underwent curatively intended chemoradiation (CRT).

## Patients and methods

### Patients and treatment

The cohort in this study included 31 consecutive cervical cancer patients treated between 2010 and 2016 with definitive CRT at the Department of Radiation Oncology at the Klinikum rechts der Isar, Munich. A total of 24 patients without paraaortic RT was included in the presented dosimetric analysis. Details of the whole cohort are listed in Table 1.

**Table 1:** Patient characteristics

	No. patients (%)
Number patients	31 (100%)
Age years, median (range)	54 (25–78)
Histology	
Squamous cell carcinoma	27 (87%)
Adenocarcinoma	4 (13%)
FIGO stage	
IIA	2 (6.5%)
IIB	6 (19.4%)
IIIA	1 (3.2%)
IIIB	5 (16.1%)
IVA	5 (16.1%)
IVB	12 (38.7)
TNM stage	
T1	4 (12.9%)
T2	16 (51.6%)
T3	6 (19.4%)
T4	5 (16.1%)
N0	4 (12.9%)
N1	24 (77.4%)
Nx	3 (9.7%)
Total radiation dose Gy, mean	
Tumor region	54,5
Lymphatic drainage	51,5
Pathologic lymph nodes	55,5
Paraortical lymph node irradiation	
No	24 (77.4%)
Yes	7 (22.6%)
Boost to pathological lymph nodes	
No	18 (58.0%)
Yes	13 (42.0%)
RT technique	
IMRT with Helical tomotherapy	6 (19.3%)
Step-and-shoot IMRT	2 (6.5%)
VMAT	23 (74.2%)
Acute toxicity (CTCAE and RTOG criteria)	
Leukopenia	
Grade 1	3 (9.7%)
Grade 2	4 (13.0%)
Grade 3	17 (54.8%)
Grade 4	2 (6.5%)
Anemia	
Grade 1	8 (25.8%)
Grade 2	13 (42.0%)
Grade 3	5 (16.1%)
Grade 4	0
Thrombocytopenia	
Grade 1	3 (9.7%)
Grade 2	2 (6.5%)
Grade 3	3 (9.7%)
Grade 4	0

*AUC* area under the curve, *CTCAE* common terminology criteria for adverse events, *FIGO* international federation of gynecological oncology, *IMRT* intensity modulated radiation therapy, *RT* radiation therapy, *RTOG* radiation therapy oncology group, *VMAT* volumetric intensity modulated arc therapy

Some patients had relevant comorbidities. Seven patients suffered from concomitant nephrological diseases (renal insufficiency: *n* = 3, a nonfunctioning kidney: *n* = 1, partial kidney resection due to a cyst: *n* = 1, nephrolithiasis: n = 1, history of acute renal failure: *n* = 1) and 3 patients suffered from concomitant cardiological diseases (atrial fibrillation: *n* = 1, history of after PTCA: n = 1, undefined heart disease: *n* = 1).

Simultaneously with percutaneous radiotherapy, 29 patients received chemotherapy. Twenty-two patients received cisplatin 40 mg/m^2^ (2 cycles (*n *= 1), 3 cycles (*n* = 6), 4 cycles (*n* = 5), 5 cycles (n = 4) and 6 cycles (n = 6)]; one patient was switched to carboplatin AUC2 after 4 cycles due to a low glomerular filtration rate (GFR). One patient each received only carboplatin AUC2 (4 cycles), vinorelbine 40 mg/m^2^ (7 cycles) and vinorelbine 70 mg/m^2^ (4 cycles). Four patients received vinorelbine 15 mg/m^2^ (5 cycles (*n* = 2) and 6 cycles (*n* = 2) due to low renal function. Two patients refused chemotherapy.

### Technical details

All patients underwent planning computed tomography in supine position with intravenous contrast medium and 5 mm slices. The clinical target volume (CTV) was based on common target volume delineation guidelines (RTOG) and consisted of the uterus, cervix, parametria, upper half of the vagina if involved and regional lymph nodes. The paraaortic nodes were included if radiographically involved and a planning target volume (PTV) was defined using an isotropic margin around the CTV of 10 mm adopted to anatomical structures. Six patients were treated in IMRT technique with helical tomotherapy, 23 patients had a VMAT and two patients had a step-and-shoot IMRT plan.

Twenty-seven patients underwent curative definitive therapy, two patients received a palliative concept, one patient switched from palliative to curative treatment and one patient from a curative to a palliative concept. The dose to the tumor region was 50.4 á 1.8 Gy (*n* = 22) or 45 á 1.8 (*n* = 8). One patient received once a single fraction of 2 Gy and then 1.8 Gy to a total dose of 50.6 Gy. In 8 patients the paraoartical lymph nodes were included in the treatment plan. Thirteen patients received a boost to pathological lymph nodes. The mean value of the total radiation dose was 54.5 Gy, for the lymphatic drainage 51.5 Gy and for the pathologic lymph nodes 55.5 Gy.

Brachytherapy (BT) was performed in 24 patients (mean value 23.7 Gy) after external beam RT based on MRI planning encompassing the residual tumor at the time of BT planning with single doses of 5 (*n* = 5) or 7 Gy (*n* = 18). One patient received twice BT in PDR-AL technic with 0.4 Gy/h with a total dose of 16 Gy/session. Dose prescription and dose constraints were followed according to GEC-ESTRO criteria [11].

### Definition of bone marrow-relevant substructures

The pelvic bone included the os sacrum, os ilium, os ischii and os pubis. The femoral head included the upper femur, the bone cortex was also included. The lumbar spine was not included. The dosimetric parameters were defined as the volumes of a definite radiation dose, such as Vx means the total volume of the bone that received the radiation dose of X Gy. In addition, the analyzed parameters included the V5, V10, V20, V30, V40, V50, and the minimal, maximal and mean doses. Patients with paraaortic RT were excluded from dosimetric analyses of BM subvolumes.

### Toxicity assessment

The hematological data, including WBC, hemoglobin concentration and platelet count were recorded from laboratory studies performed under CRT. All available information about the relevant acute therapy side effects according CTCAE and RTOG criteria (nausea, leucopenia, anemia, thrombopenia, loss of weight, diarrhea, skin reaction, fatigue, vaginal mucositis, urological side effects) were assembled out of the patient files. For the correlation of the blood levels the nadir of WBCs, hemoglobin levels and thrombopenia during CRT were analyzed.

### Statistics

The overall survival (OS) and progression-free survival (PFS) were estimated according to Kaplan–Meier. Pearson correlation coefficient (PCC) was calculated for the correlation of absolute blood levels and PB and FH constraints. Spearman correlation coefficient as well as logistic regression and scatter plots were used for the correlation of blood levels according to CTCAE.

## Results

### Overall survival and progression-free survival

The mean OS was 30 months (range 3–82 months) with 11 deaths reported. PFS was defined as absence of loco-regional or distant metastatic failure. The PFS was 17.4 months (range 0–63 months). Referring to RECIST criteria 11 patients had a complete response after CRT. Nine patients were in partial remission; two patients had a stable disease and seven patients had a progressive disease with distant metastases.

### Hematologic toxicity

Regarding information about the relevant acute and late therapy side effects three patients had a grade 4 acute toxicity according to CTCAE criteria for leukopenia (*n* = 2) and diarrhea (*n* = 1). Grade-3 acute toxicity existed for leukopenia (*n* = 17), anemia (*n* = 5), thrombopenia (*n* = 3), diarrhea (*n *= 2), nausea (*n* = 6), vaginal bleeding (*n* = 1) and dysuria (*n* = 2). Except for one patient suffering from grade-3 loss of weight (according to late toxicity RTOG criteria) only grade 1 and 2 late toxicities occurred.

### Correlation of blood levels and PB and FH constraints

Comparing the absolute blood levels with the dose volumes of both FH and PB the data showed a significant correlation between WBC and the median dose of the FH and the median dose, V_30Gy_, V_40Gy_ and V_50Gy_ of the PB (Table 2). A correlation between the toxicity grade of anemia and mean dose, maximum dose and V_5Gy_ of the PB was found. Counting the highest grade of HT of all three blood levels of each patient, significant correlations were found for the mean and median dose, V_30Gy_, V_40Gy_ and V_50Gy_ of the PB. Further dosimetric details of BM subsites are presented in Table 3.

**Table 2:** Spearman’s correlation coefficient of blood levels according CTCAE and dose constraints of pelvic bone and femoral heads

DVH parameters	Hemoglobin		WBC		Platelets		worst case	
	Correlation coefficient	*p* value	Correlation coefficient	*p* value	Correlation coefficient	*p* value	Correlation coefficient	*p* value
Right femoral head								
Mean dose	− 0.199	0.375	− 0.529*	**0.011**	− 0.286	0.196	− 0.458*	**0.032**
Maximum dose	− 0.355	0.105	− 0.442*	**0.039**	− 0.285	0.199	− 0.672**	**0.001**
Median dose	− 0.029	0.897	− 0.475*	**0.025**	− 0.246	0.270	− 0.349	0.111
Volume	0.337	0.126	− 0.125	0.580	− 0.054	0.811	− 0.062	0.782
V_5Gy_	0.001	0.997	− 0.585**	**0.004**	− 0.472*	**0.027**	− 0.447*	**0.037**
V_50Gy_	− 0.309	0.173	− 0.525*	**0.014**	− 0.430	0.052	− 0.734**	**0.000**
Left femoral head								
Mean dose	-0.220	0.302	− 0.387	0.062	− 0.173	0.418	− 0.342	0.101
Maximum dose	-0.310	0.141	− 0.373	0.072	− 0.308	0.143	− 0.489*	**0.015**
Median dose	-0.190	0.373	− 0.406*	**0.049**	− 0.175	0.413	− 0.271	0.199
Volume	0.367	0.078	0.016	0.939	0.147	0.494	− 0.054	0.803
V_5Gy_	-0.036	0.867	− 0.558**	**0.005**	− 0.401	0.052	− 0.452*	**0.027**
V_50Gy_	-0.265	0.221	− 0.393*	**0.063**	− 0.297	0.169	− 0.573**	**0.004**
Pelvic bone								
Minimum dose	− 0.258	0.224	0.208	0.330	− 0.116	0.589	− 0.031	0.885
Mean dose	− 0.489*	**0.015**	− 0.177	0.407	− 0.259	0.222	− 0.468*	**0.021**
Maximum dose	− 0.445*	**0.029**	− 0.124	0.563	− 0.209	0.326	− 0.310	0.141
Median dose	− 0.375	0.071	− 0.485*	**0.016**	− 0.365	0.079	− 0.514**	**0.010**
Volume	0.381	0.066	0.110	0.610	0.099	0.645	− 0.107	0.618
V_5Gy_	− 0.300	0.154	0.310	0.140	− 0.120	0.575	0.005	0.981
V_10Gy_	−0.302	0.151	0.123	0.566	− 0.042	0.846	− 0.089	0.680
V_15Gy_	− 0.223	0.294	0.112	0.603	− 0.084	0.697	0.084	0.695
V_20Gy_	− 0.288	0.173	− 0.115	0.593	− 0.244	0.251	− 0.126	0.556
V_30Gy_	− 0.311	0.139	− 0.416*	**0.043**	− 0.305	0.148	− 0.446*	**0.029**
V_40Gy_	− 0.385	0.063	0.000*	**0.012**	− 0.400	0.053	− 0.528**	**0.008**
V_50Gy_	− 0.445*	**0.034**	− 0.498*	**0.016**	− 0.386	0.069	− 0.625**	**0.001**

Bold values represent significant results

^*^Correlations are significant at the 0.05 level (2-tailed); ** Correlations are significant at the 0.01 level (2-tailed)

*CTCAE* common terminology criteria for adverse events, *V* Volume, *WBC* white blood cells, *DVH* dose volume histogram

**Table 3:** Dosimetric analysis according to bone marrow subsite

Right femoral head (*n* = 29)	Mean [%]	St. dev [%]	minimum [%]	maximum [%]
Mean dose	19.64	4.55	12.03	31
Max. dose	47.03	5.82	34.49	61.52
Median dose	17.07	5.22	3.67	30.1
Volume [cm^3^]	114.27	23.27	42.04	149.5
V_5Gy_	95.34	8.59	68.3	100
V_50Gy_	0.56	1.59	0	6.49

left femoral head (*n* = 31)	Mean [%]	St. dev [%]	minimum [%]	maximum [%]
Mean dose	19.2	4.93	9.87	31.86
Max. dose	46.90	8.55	22.41	64.31
Median dose	17.41	6.76	9.6	41.48
Volume [cm^3^]	115.49	25.11	37.29	170.3
V_5Gy_	94.96	9.13	64.31	100
V_50Gy_	0.52	1.25	0	5.95

Pelvic bone (*n *= 29)	Mean [%]	St. dev [%]	Minimum [%]	maximum [%]
Mean dose	34.25	4.77	28.09	53.5
Max. dose	58.25	9.41	46.23	98.7
Median dose	35.62	5.45	27.59	52.8
Volume [cm^3^]	869.03	132.76	524.1	1134.9
V_5Gy_	98.94	1.97	92.9	100
V_10Gy_	97.12	3.83	88.19	100
V_15Gy_	92.29	4.8	82.51	99.08
V_20Gy_	84.3	5.58	70.6	98.01
V_30Gy_	61.97	10.27	41.44	89.46
V_40Gy_	38.08	13.23	18.9	74.66
V_50Gy_	13.3	11.63	0.09	55.83

## Discussion

In our analysis of cervical cancer patients undergoing CRT with IG-VMAT, we were able to identify a defined subset of BM dose–volume parameters that seem to correlate with different forms of HT. Notably, intermediate and higher doses (subvolumes receiving 30–50 Gy) of the PB had a negative impact on WBC and mean dose as well as lower doses (V_5Gy_) lead to higher HT in terms of anemia. Correlations of the dose–volume parameters with HT according to CTCAE by logistic regression failed to show any correlation. Scatter plots though showed an apparent correlation with a tendency to higher toxicity grades with higher radiated dose volumes so that probably a larger patient cohort is necessary to show a significance.

Previous studies have already evaluated associations between dose**–**volume metrics on BM and the development of HT, although most of them were retrospective analyses [6, 12–16]. Table 4 gives an updated overview of recent studies that examined the influence of BM dose–volume parameters and HT in cervical cancer patients undergoing (IG-)IMRT/VMAT-based CRT.

**Table 4:** Up-to-date review of studies on IG-IMRT/-VMAT-based CRT and correlation of BM dose constraints with hematotoxicity

	Type of study	Results	Limitations
Mell et al. 2017	- Prospective multicentre II trial, IMRT for unresected cervical cancer patients, IG-IMRT vs. IMRT - subgroup of patients had PET-contoured ABM - BM constraints V_10Gy_ and V_20Gy_ < 90% and < 75% were calculated	- V_10Gy_/V_20Gy_/V_30Gy_/V_40Gy_ lower with IG-IMRT and significantly less grade 3 neutropenia	- No use of VMAT, all patients underwent step-and-shoot IMRT
Williamson et al. 2022	- International phase-II/III trial (INTERTECC) - PET-based bone-marrow sparing IG-IMRT vs. standard IG-IMRT - 101 patients were enrolled on the phase II/III trial, including 29 enrolled in phase III (PET-BMS-IMRT group: 16; IMRT group: 13)	- Incidence of acute grade ≥ 3 neutropenia was significantly lower in the PET-BMS-IMRT group compared to IMRT (19% versus 54%) - No difference in post-treatment ALC by treatment group - No evidence that PET-BMS-IMRT impacted chemotherapy delivery or long-term outcomes, weak evidence of an association between pre-treatment ALC and OS	- Only 29/101 patients enrolled in phase-III - Possible bias because non-randomized phase-II patients were included in the analysis - Analysis of lymphopenia was not defined pre-hoc
Zhang et al., 2023	- Retrospective - 117 patients undergoing CRT - IMRT/VMAT	- V5, V10, V20, and V30 of BM are significantly correlated with lymphocytic toxicity	- Retrospective - Induction chemotherapy - Concomitant chemotherapy with Paclitaxel and Cisplatin - No use of FDG-PET
Chen et al., 2023	- Retrospective - 69 patients undergoing CRT - IMRT/VMAT - Division of BM into ilium (IL), lower pelvis (LP) and lumbosacral spine (LS)	- BM toxicity possibly masked by number of chemotherapy cycles: categorization into group A (3–4 cycles) and group B (5–6 cycles) - Group B: No detected correlation with dosimetric values - Group A: correlation of relative IL-V15, relative IL-V50 and absolute LP-V50 with neutrophil nadir	- Retrospective - Most patients received postoperative RT - No use of FDG-PET
This study	- Retrospective - 24 patients undergoing definite CRT without paraaortic lymph node irradiation in VMAT or IMRT technique - PB and FH BM were evaluated	- Leukopenia correlated significantly with median dose of both FH and median dose, V_30Gy,_ V_40Gy_ and V_50Gy_ of the PB - Hemoglobin levels correlated significantly with mean and maximum dose and V_5Gy_ of the PB - V_30Gy_, V_40Gy_, and V_50Gy_ of the PB correlated with the highest existing grade of HT	- Retrospective - No use of PET

*ABM* active bone marrow, *BM* bone marrow, *BMS* Bone Marrow Sparing, *cc* cubic centimeter, *CRT* chemoradiation, *CTCAE* common toxicity criteria, *DVH dose* volume histogram, *IMRT* intensity-modulated radiation therapy, *IG* image-guided, *HT* hematotoxicity, *ALC* absolute lymphocyte count, *PBM* pelvic bone marrow, *PET* positron emission tomography, *V* volume, *VMAT* volumetric-modulated radiation therapy

Zhang and colleagues retrospectively examined outcomes of 117 patients undergoing CRT with IMRT/VMAT. The V_5Gy_, V_10Gy_, V_20Gy_, and V_30Gy_ of BM correlated significantly with lymphocytic toxicity. A strong limitation was the uncommon use of induction chemotherapy and a concomitant chemotherapy with Paclitaxel and Cisplatin, which makes comparison with other work considerably more difficult. The group of Chen et al. performed a retrospective analysis of 69 patients undergoing CRT with IMRT/VMAT. They subdivided the BM into ilium (IL), lower pelvis (LP) and lumbosacral spine (LS) and evaluated dosimetric predictors of BM toxicity. The authors only found a difference in patients who had more chemotherapy cycles (5–6 vs. 3–4), so one can assume that BM toxicity was possibly masked by the amount of BM-suppressing cytostatic therapy. In the group with less CT cycles the relative IL-V_15_, relative IL-V_50_ and absolute LP-V_50_ correlated with the neutrophil nadir.

Although most of this work has defined the bony structures as BM equivalent, some authors proceed methodologically differently. These take positron emission tomography (PET) as the basis of the BM definition to define active BM [13, 17]. The FDG uptake in PET/CT scans can define active BM regions and correlates with the number of hematopoietic marrow cells [7, 18]. The uptake within the radiation field decreases after treatment and is variable across irradiated bone sites, which indicates myelosuppression [18]. In conjunction with the use of modern radiation techniques such as IMRT and VMAT, this methodology offers greater accuracy when trying to spare relevant BM subvolumes in order to reduce toxicity.

The group of Mell and colleagues initiated a remarkable prospective multicenter phase II trial, which examined for the first time the dosimetric impact and relevance of PET-based BMS-IG-IMRT [13]. The BM constraints used were V10Gy and V20Gy < 90% and < 75%, respectively. The results showed that V_10Gy_/V_20Gy_/V_30Gy_/V_40Gy_ were considerably lower with IG-IMRT and led to significantly less grade-3 neutropenia. The same group was able to set up the international phase-II/-III trial (INTERTECC) that accrued 101 patients, which is also the most relevant study in this field today[19]. The question of the study was whether PET-based bone marrow sparing IG-IMRT reduces toxicity compared to standard IG-IMRT. The results showed a significant lower incidence of acute grade ≥ 3 neutropenia in the PET-BMS-IMRT group as compared to IMRT (19% versus 54%), but no difference in post-treatment ALC by treatment group. Furthermore, according to the authors there was no evidence that PET-BMS-IMRT affected chemotherapy delivery or long-term outcomes. However, only 29 out of 101 patients were finally enrolled in the phase-III part and a possible bias cannot be excluded.

Although the studies by Zhang, Chen and Mell showed an effect on neutropenia or lymphopenia even at low doses (V10Gy, V20Gy, IL-V15), we were only able to detect a correlation in our cohort at higher doses (V30-50). The fact that a correlation between Hb value and V5Gy, mean and maximum dose was found in the PB in our study was not seen in any of the other studies, so that we must assume that this is most likely an artifact.

Several studies have now been able to show that the BM dose can safely be reduced by IG-VMAT or, at best, the targeted use of BM-protective constraints leads to lower toxicities. Nevertheless, the clinical relevance of these efforts has not yet been proven beyond doubt. A recent meta-analysis on this topic was also able to confirm that protecting the pelvic bones can lead to a reduction in the bone marrow dose and HT [20]. At the same time, however, the authors admitted that a clinical benefit of these measures in the broad is still pending. Despite the lack of clear evidence of superiority, sparing of the relevant BM structures can generally be achieved through modern radiation planning and without compromising target volume coverage or increasing other toxicities (gastrointestinal, genitourinary). Based on our own data and the literature discussed, we prepared a tabular summary in which useful BM constraints from previous studies are suggested (Table 5) [12, 14, 19, 21, 22].

**Table 5:** Dose constraints for bone marrow

Radiation technique	IMRT/VMAT, IGRT		
BM definition	FDG-PET (above SUV mean) or Pelvic bone (± substructure), femoral heads, lumbosacral spine		
	Anatomical region	Constraint	References
DVH parameters			
	Lower pelvis	V_5Gy_ < 95%	(Kumar et al. [12])
	Pelvic BM	V_10Gy_ < 90%	(Mell et al. [13])
		V_20Gy_ < 80%	(Albuquerque et al. [21])
		V_30Gy_ < 65%	(Kumar et al. [12])
	Iliac crests	Mean dose < 31 Gy	(Kumar et al. [12])
	ABM (FDG-PET)	V_40Gy_ < 738 ml	(Zhou et al. [14])

*ABM* active bone marrow, *BM* bone marrow, *cc* cubic centimeter, *CRT* chemoradiation, *CTCAE* common toxicity criteria, *DVH* dose–volume histogram, *IGRT* image-guided radiotherapy, *IMRT* intensity-modulated radiation therapy, *HT* hematotoxicity, *PBM* pelvic bone marrow, *V* volume, *VMAT* volumetric-modulated radiation therapy

Finally, our study had several limitations due to the small patient group and the retrospectively gained data and needs a prospective cohort to affirm these findings. Owing to the low patient number, a logistic regression model to correlate the different CTCAE toxicity grades with the dose volumes was not robust enough.

However, the results of this and previous studies show that HT correlates with distinct subvolumes in FH and PB.

## Data Availability

No datasets were generated or analysed during the current study.

## Abbreviations

HT: Hematotoxicity
ABM: Active bone marrow
BM: Bone marrow
BMS: Bone marrow sparing
cc: Cubic centimeter
CRT: Chemoradiation
CTCAE: Common toxicity criteria
DVH: Dose–volume histogram
IMRT: Intensity-modulated radiation therapy
IG: Image-guided
HT: Hematotoxicity
ALC: Absolute lymphocyte count
PB: Pelvic bone
PET: Positron emission tomography
VMAT: Volumetric-modulated radiation therapy
